# Supplementary material for: Identification of photoperiod-induced specific miRNAs in the adrenal glands of Sunite sheep (Ovis aries)
Source: Front Vet Sci. 2022 Jul 22;9:888207. doi: 10.3389/fvets.2022.888207 (PMC9354845; doi:10.3389/fvets.2022.888207)
Supplement: Supplementary file 1 [file Data_Sheet_1.ZIP › Supplementary file/miRDeep2 file.zip!/miRDeep2 file/Know_Structure/SPLP42A2/oar-mir-376d.pdf]

miRBase precursor : oar-mir-376d
Total read count : 138
oar-miR-376d read count : 133
remaining reads : 5

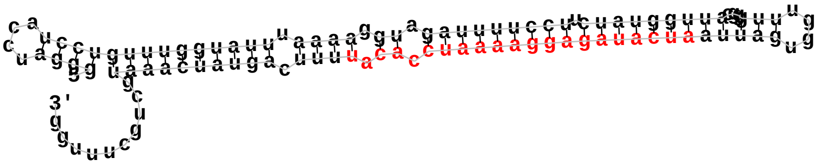

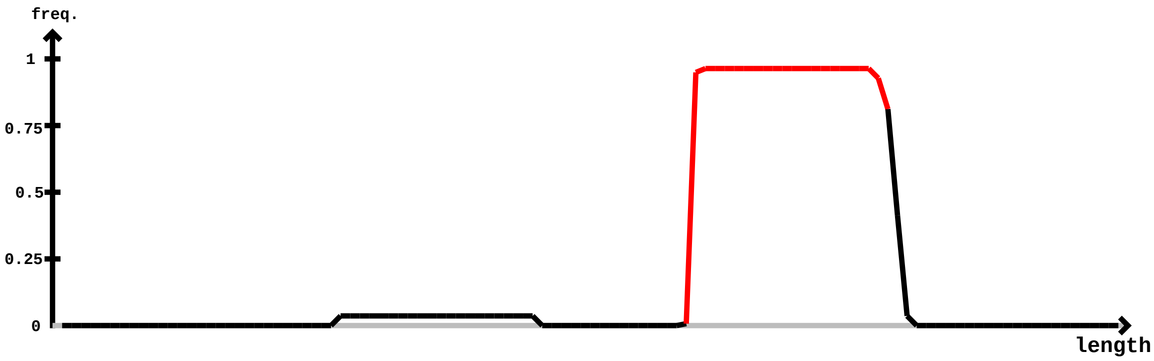


```
                                                                      oar-miR-376d
5'- gggauccauccuguuugguauuuaaaagguagauuuuccuucuaugguuacggguuuggugauuaaucauagaggaaaauccacauuuucaguaucaaaugcugcuuugg   -3'    exp
    ((((....))))((((((((((.((((.((.(((((((((.(((((((((...(((....))))))))))))))))))))).)).)))).))))))))))..........    reads    mm    sample
    ............................guagauuuuccuucuaugguu.............................................................    5        0     seq
    ................................................................aaucauagaggaaaauccac..........................    1        0     seq
    .................................................................aucauagaggaaaauccac..........................    4        0     seq
    .................................................................aucauagaggaaaauccaca.........................    16       0     seq
    .................................................................aucauagaggaaaauccacau........................    55       0     seq
    .................................................................aucauagaggaaaauccacauu.......................    51       0     seq
    .................................................................aucauagaggaaaauccacauuu......................    4        0     seq
    ..................................................................ucauagaggaaaauccacauu.......................    1        0     seq
    ..................................................................ucauagaggaaaauccacauuu......................    1        0     seq
```
